# Proteomic analysis of plasma unravels dynamic pathways and potential biomarkers indicating disease stages following *Mtb* infection

**DOI:** 10.1128/msystems.00616-25

**Published:** 2025-07-30

**Authors:** Zonglei Zhou, Jie Tian, Yuying He, Haiyan Xiong, Weibing Wang

**Affiliations:** 1 School of Public Health, Shanghai Institue of Infectious Disease and Biosecurity, Fudan University 12478, Shanghai, China; 2 Shanghai Pudong Hospital, Fudan University Pudong Medical Center 542170, Shanghai, China; 3 Institute of Tuberculosis Control and Prevention, Guizhou Provincial Center for Disease Control and Prevention, Guiyang, China; 4 Key Laboratory of Public Health Safety of Ministry of Education, Fudan University 12478, Shanghai, China; National Institutes of Health, National Institute of Allergy and Infectious Diseases, Bethesda, Maryland, USA

**Keywords:** tuberculosis, latent *Mtb *infection, plasma proteomics, differentially expressed proteins, diagnostic biomarkers

## Abstract

Tuberculosis (TB) stages depend greatly on the interaction between *Mycobacterium tuberculosis* (*Mtb*) and the host response. As a non-negligible source of active tuberculosis (ATB), individuals with latent *Mtb* infection (LTBI) are insidious and hard to be detected due to limited biomarkers. Further insight into the pathogenic mechanisms associated with heterogeneous clinical outcomes after *Mtb* infection benefits the prevention and control of TB epidemics. Therefore, this study employed four-dimensional data-independent acquisition to quantify the expression level of plasma proteins from healthy controls (HC), LTBI, and ATB, with 15 participants in each group. Key proteins related to TB stages were further assayed using parallel reaction monitoring (PRM) for validation in an independent cohort, with a sample size of 20 individuals per group. Differential abundance analyses showed a notable increase in the number of differentially expressed proteins (DEPs) following *Mtb* infection, mainly involving carbohydrate catabolism, metabolism of cholesterol and lipid, immune response and inflammation, and complement and coagulation cascades. Protein-protein interaction network suggested similar functional enrichment patterns as aforementioned for core DEPs. Weighted gene co-expression network analyses identified six modules, among which the brown module significantly correlated with TB stages. Hub proteins in the brown module were enriched in lipid metabolism and acute-phase response. Furthermore, PRM protein quantification revealed that complement factor H (area under receiver operating characteristic curves [AUC] = 0.708) had a better performance in differentiating LTBI and HC than C4B (AUC = 0.625), while C4B (AUC = 0.917), MBL2 (AUC = 0.887), and SAA1 (AUC = 0.875) were helpful in differentiating ATB and HC. Also, SAA1 (AUC = 0.917) and matrix Gla protein (AUC = 0.905) favored the discrimination of ATB from LTBI. Our work probes into the hub plasma proteins and pathways associated with disease stages following *Mtb* infection, providing critical implications on the diagnosis of TB stages.

**IMPORTANCE:**

Distinct prognostic outcomes following *Mycobacterium tuberculosis* (*Mtb*) infection result from host-pathogen interactions, while the response mechanisms underlying such heterogeneous phenotypes are far from understood. Through four-dimensional data-independent acquisition and parallel reaction monitoring, our study linked specific plasma proteomic profiles to various tuberculosis (TB) stages and corroborated relevant pathways under various disease conditions. Of identified core proteins, complement factor H formed a diagnostic classifier that distinguished latent *Mtb* infection from healthy controls with good performance, and we also identified C4B, MBL2, SAA1, and matrix Gla protein as potential proteomic signatures of active tuberculosis. Additionally, this study further highlighted the critical role of carbohydrate and lipid metabolism, immunological responses, and blood coagulation in TB pathogenesis. Taken together, our findings feature a dynamic landscape of plasma proteome following *Mtb* infection and provide additional evidence on plasma biomarkers for TB diagnosis.

## INTRODUCTION

In 2023, tuberculosis (TB) contributes to the world’s leading cause of death from a single infectious agent, outweighing the number of deaths attributed to HIV/AIDS (1). Globally, individuals infected by *Mycobacterium tuberculosis* (*Mtb*) account for 25% of the whole population, and most are absent from symptoms of active tuberculosis (ATB) and remain in a state of persistent immune response to *Mtb* antigen stimulation, termed as latent *Mtb* infection (LTBI) (2, 3). Prior research has suggested that people at the LTBI stage have a 5%–15% life-time risk of progressing to ATB (4), and most ATB cases occur within 2 years after infection (5–7), with median time of <1 year (8), implicating early immune events as key determinants of prognosis. Notably, even with high adherence to preventive therapy, around 25–33 individuals with LTBI need to take treatments for latent tuberculosis to prevent one person from progressing to ATB, suggesting the great costs and efforts in controlling the TB epidemic (9, 10).

Currently, diagnostic methodologies to identify those infected with *Mtb* predominantly encompass the tuberculin skin test and interferon-gamma release assays (IGRAs). A recent study targeting IGRAs demonstrated its excellent prognostic ability in predicting individual risk of developing TB, while IGRAs appear to have decreased detection sensitivity among immunosuppressive populations (11, 12). Also, these tools are not fit for large-scale screening of *Mtb*-infected individuals in light of their tedious and time-consuming procedures (13). For individuals with LTBI, the pathogen is primarily contained within lung granulomas or draining lymph nodes, making direct detection of the bacterium nearly impossible (14). Longitudinal whole blood transcriptomic analyses suggested that TB pathogenesis involves sequential modulation of immunological processes (15). Host signals in the blood, such as inflammatory markers, have been shown to reflect host-pathogen interactions and can be used to identify individuals at a great risk of developing TB (16, 17). Despite the good performance of these RNA-based biomarkers, the measurement of plasma proteins is more practical for the development of timely tests and large-scale application in populations. Furthermore, it is well acknowledged that proteins play a crucial role in maintaining bodily functions through various signaling pathways, and explorations on the blood proteome landscape can offer a more reliable and direct perspective to elucidate the host response against *Mtb* infection.

To date, several studies have suggested potential host plasma protein biomarkers for *Mtb* infection. Differential protein profiles have identified an array of TB signatures, like CD14 (18), *C*-reactive protein (CRP) (19), FCGR3B (18), GBP1 (20), ferritin (21), HO-1 (22), and TIMP-4 (23). As for combined biomarkers, Chegou et al. (24) proposed a seven-marker signature (CRP, transthyretin, IFN-γ, complement factor H [CFH], APOA1, IP-10, and SAA) for the differentiation between confirmed TB, probable TB, and non-pulmonary TB. Employing serum samples from TB and non-TB, De Groote et al. (25) validated the six-marker signature (tryptophan-tRNA ligase [SYWC], kallistatin, C9, gelsolin [GSN], testican-2, and aldolase C) as a reliable classifier. Similarly, a nine-protein signature was established to distinguish TB from other diseases (26). Notably, diagnoses based on numerous signatures could result in higher costs and may bring great difficulty in design and manufacture, precluding the implementation of large-scale point-of-care tests. Despite existing findings, there is a lack of comprehensive and systematic evidence profiling the differential plasma proteins associated with various clinical outcomes following *Mtb* infection, particularly the pathways responsible for LTBI that are far from understood. Furthermore, there exists great heterogeneity in diagnostic features presented by these studies, and the biomarkers in discriminating LTBI from healthy controls (HC) are limited by far.

Thus, our study endeavors to identify and validate biomarkers capable of differentiating patients at different stages following *Mtb* infection by conducting a comparative analysis of plasma proteome signatures. Furthermore, this study aims to investigate critical pathways and hub proteins involved in *Mtb* infection, thereby providing helpful evidence over TB pathogenesis.

## MATERIALS AND METHODS

### Participants and sample collection

The current study was implemented in the Traditional Chinese Medicine Hospital of Qingzhen City, Guizhou Province. Patients with active pulmonary TB were considered as ATB cases if they indicated: (i) positive sputum culture for *Mtb*; (ii) positive nucleic acid test by GeneXpert MTB/RIF; (iii) chest radiographic evidence suggesting *Mtb* infection. Individuals with all negative tests and no clinical symptoms of ATB were further subjected to perform *Mtb*-specific IGRAs, which have been consistently reported as a reliable tool to discriminate individuals with LTBI from HC (12, 27–29). Participants were excluded if meeting one of the following conditions: (i) age <18 years; (ii) diagnosed with immune dysfunctions, metabolic diseases, cancer, and other pathogen infections; (iii) taking anti-TB treatments; (iv) extrapulmonary TB; (v) refusal to sign informed consents. Subsequently, 5 mL of peripheral blood was collected using an EDTA anticoagulant blood collection tube for each of 105 individuals (35 per group) at different stages, with no distinct difference in age and gender between groups (Table S1). Blood samples were centrifuged at 3,000 g for 10 minutes to extract plasma, which was then preserved at −80°C for further assays. Samples were randomly assigned to the derivation cohort (45 participants with 15 in each group) and validation cohort (60 participants with 20 in each group) in a ratio of 3:4 (Table S1). The protocol of our study was approved by the Medical Research Ethics Committee of Fudan University School of Public Health (IRB#2021-11-0929), and written informed consent was obtained from all participants.

### Protein abundance quantification

To reduce the impacts on quantitative determination exerted by high-abundance proteins, IgG and albumin were depleted using the Albumin/IgG removal kit (Thermo Scientific). Total protein concentration was obtained by bicinchoninic acid protein assay kit (Thermo Scientific). Next, protein samples were reduced with 10 mM tris(2-carboxyethyl)phosphine (TCEP) and alkylated with 40 mM iodoacetamide, and we digested proteins through overnight trypsinization. Degraded peptides were dissolved using 0.1% trifluoroacetic acid and desalted with hydrophilic-lipophilic-balanced (HLB) solid-phase extraction columns. Then, a spectral library was generated using data-dependent acquisition (DDA) with a Vanquish Flex UHPLC system (Thermo Scientific) as previously described (30, 31). For four-dimensional data-independent acquisition (4D-DIA), each sample was subjected to protein trypsinization and peptide desalting as described above, and then purified peptides were subjected to liquid chromatography coupled to mass spectrometry (LC-MS) instrumentation consisting of timsTOF Pro2 (Bruker). The abundance of targeted proteins in the validation cohort was quantified by parallel reaction monitoring (PRM) using a Q-Exactive HF-X mass spectrometry system (Thermo Scientific).

### MS data analyses

In this study, a previously generated deep-fractionated DDA library was used in the plasma quantification of 4D-DIA data. Specifically, with DDA data files as the input, Spectronaut software (version 14.0) was employed to search against the UniProtKB human database (32) to create a digital library of spectra for the subsequent 4D-DIA analyses. Afterward, the 4D-DIA MS data were searched against the above spectral library to obtain the quantitative abundance of each protein. The search parameters were presented as follows: (i) carbamidomethyl [C] and oxidation [M] were selected as fixed modification and variable modification, respectively; (ii) the tolerance of parent ions was set to 10 ppm and 0.02 Da for fragmented ions; (iii) maximum missed cleavage sites ≤2; (iv) false discovery rate <1% at both peptide and protein level; (v) peptide confidence ≥99%.

### Differential abundance analyses

Before formal analyses, proteins with valid values >80% in at least one group were kept for the downstream analyses, and the remaining missing values were imputed using the random forest method with DEP2 package (33). Accordingly, we detected a total of 3,798 proteins in the plasma of 45 samples, and the removal of those with missing values greater than the predefined threshold resulted in a data set comprising 2,458 proteins. Then, proteins with high abundance were further excluded from the following analyses.

We employed the DEP2 package in R software to compare the proteomic profile between groups. In brief, protein intensities were log-normalized using the variance-stabilizing transformation method, followed by between-group comparisons of proteomic profiles with the test_diff function. The ascertainment of differentially expressed proteins (DEPs) requires a fold change (FC) in protein abundance of either less than 0.8 or greater than 1.2 in combination with a *P*-value < 0.05. The heatmap and volcano plots were generated to delineate protein expression features of DEPs between groups.

### Functional annotation and enrichment analyses

We predicted the function of the interested proteins using the biological processes (BP) of gene ontology (GO) annotation and involved pathways using Kyoto Encyclopedia of Genes and Genomes (KEGG) analyses. The aforementioned analyses were carried out using the ClusterProfiler package in R software, and the adjusted *P*-value for each item was obtained using the Benjamini-Hochberg method.

### Protein-protein interaction analyses

The interaction of interested proteins was explored utilizing the Search Tool for the Retrieval of Interacting Genes/Proteins (STRING, version 12.0) database (34), with the species set as “*Homo sapiens*” and interaction confidence greater than 0.40. The resulting protein-protein interaction (PPI) networks were plotted using Cytoscape software (version 3.10.0). Furthermore, we employed the maximum clique centrality (MCC) algorithm embedded in the Cytohubba plugin of Cytoscape software to identify core proteins that may play a crucial role in host response against the bacterial infection (35).

### Construction of gene co-expression network

To probe the co-expression relationship between proteins, a scale-free network was built by weighted gene co-expression network analyses (WGCNA) (36). First, we employed the pickSoftThreshold function of the WGCNA package in R software to determine a suitable soft-thresholding power that can enable the scale-free topology fit index ≥0.85. Next, based on the protein expression matrix, the blockwiseModules function was used to construct co-expression modules with the power identified in the previous step and a minimum module size of 10. We computed the correlation of modules with disease stages to dissect core modules, in which proteins were further analyzed by GO annotation and KEGG enrichment, as well as PPI analyses. Additionally, we identified hub proteins in modules significantly associated with disease conditions using the MCC method.

### Statistical analyses

One-way analysis of variance (ANOVA) was performed to test the difference in age between groups, and *χ*^2^ test was utilized for the comparison of gender distribution across samples. To assess the accuracy of biomarkers in discriminating different infection stages, receiver operating characteristic (ROC) curves were plotted for several key proteins. Furthermore, we calculated the area under ROC curves (AUC) and inferred the corresponding 95% confidence interval (CI) using the DeLong method. All statistical analyses were carried out by R software (version 4.3.1), with the significance threshold set as a two-sided *P* < 0.05.

## RESULTS

### Overall quantification of plasma proteins

In this study, the number of detected proteins for each sample ranged from 2,217 to 2,379, with a median of 2,322 (Fig. S1A). Of the total 2,458 proteins, 1,424 (57.93%) can be identified in all samples, and major proteins had the molecular weight of 10–70 kDa, with the counts decreasing by molecular weight (Fig. S1B and C). The length of the fragmented peptide was mainly distributed from 8 to 20 amino acids, which is in line with the general rule of trypsin enzymatic hydrolysis and higher-energy collisional dissociation (HCD) fragmentation (Fig. S1D). Proteins with lower abundance were more prone to have missing values (Fig. S1E), consistent with prior reports (37, 38). We imputed missing values using a random forest algorithm, and to evaluate whether data imputation may introduce biases in protein expression data, the distribution was compared to that of the original data. The results showed similar distribution between the two data sets, indicating reasonable data imputation implemented in the present study (Fig. S1F). Furthermore, the coefficient of variation (CV) for protein abundance was low in HC, LTBI, and ATB, and we found that CV values were similar between HC and LTBI, but were relatively higher in ATB compared to the formers (Fig. S1G).

### Impacts of *Mtb* infection on plasma proteomic profiles

**Fig 1:**
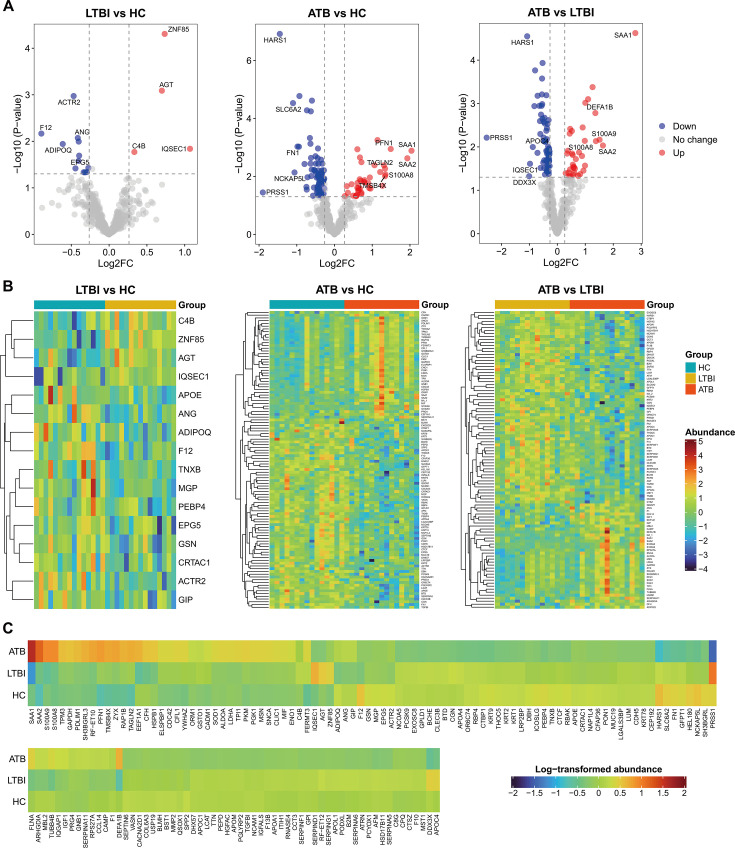
Differential expression profiles of plasma proteins from HC, LTBI, and ATB. (**A**) Volcano plot presents the DEPs between different disease stages, with upregulated proteins colored in red and downregulated proteins in blue. (**B**) Heatmap shows the abundance of dysregulated plasma proteins for each participant. (**C**) Heatmap delineates the average abundance of proteins differentially expressed in at least one group pair. Upregulated DEPs refer to proteins that have a fold change in abundance of greater than 1.2 and *P*-value < 0.05. Downregulated DEPs refer to proteins that have a fold change in abundance of less than 0.8 and *P*-value < 0.05.

To better profile the low-abundance plasma proteins, we further manually excluded immunoglobulin fragments and unknown proteins, thereby obtaining 456 proteins for further bioinformatics analysis (Table S2). In comparison with the HC group, differential abundance analyses revealed 4 upregulated proteins and 12 downregulated proteins in LTBI, as well as 39 upregulated proteins and 69 downregulated proteins in ATB (Fig. 1A and B; Fig. S2 and Table S3). When compared to LTBI, there were 35 proteins significantly enriched in individuals at the ATB stage, while 64 proteins were significantly depleted (Fig. 1A and B; Fig. S2 and Table S3). For the DEPs in HC and LTBI compared to ATB, hierarchical clustering analysis grouped upregulated proteins and those downregulated into distinct clades, suggesting favorable performance in differentiating these disease conditions (Fig. 1B). Nevertheless, the DEPs were not well clustered for the comparison group of LTBI and HC, possibly attributable to insufficient DEPs between groups. Of note, ATB exhibited an obvious increase in acute-phase proteins, like SAA1, SAA2, S100A9, S100A8, and HSPB1 (39–41). Also, several proteins involved in complement activation and inflammation were also detected, including MBL2, CFH, MIF, CCL14, SERPING1, TMSB4X, and CDH5 (Fig. 1C).

### GO and KEGG enrichment of DEPs in plasma related to TB

**Fig 2:**
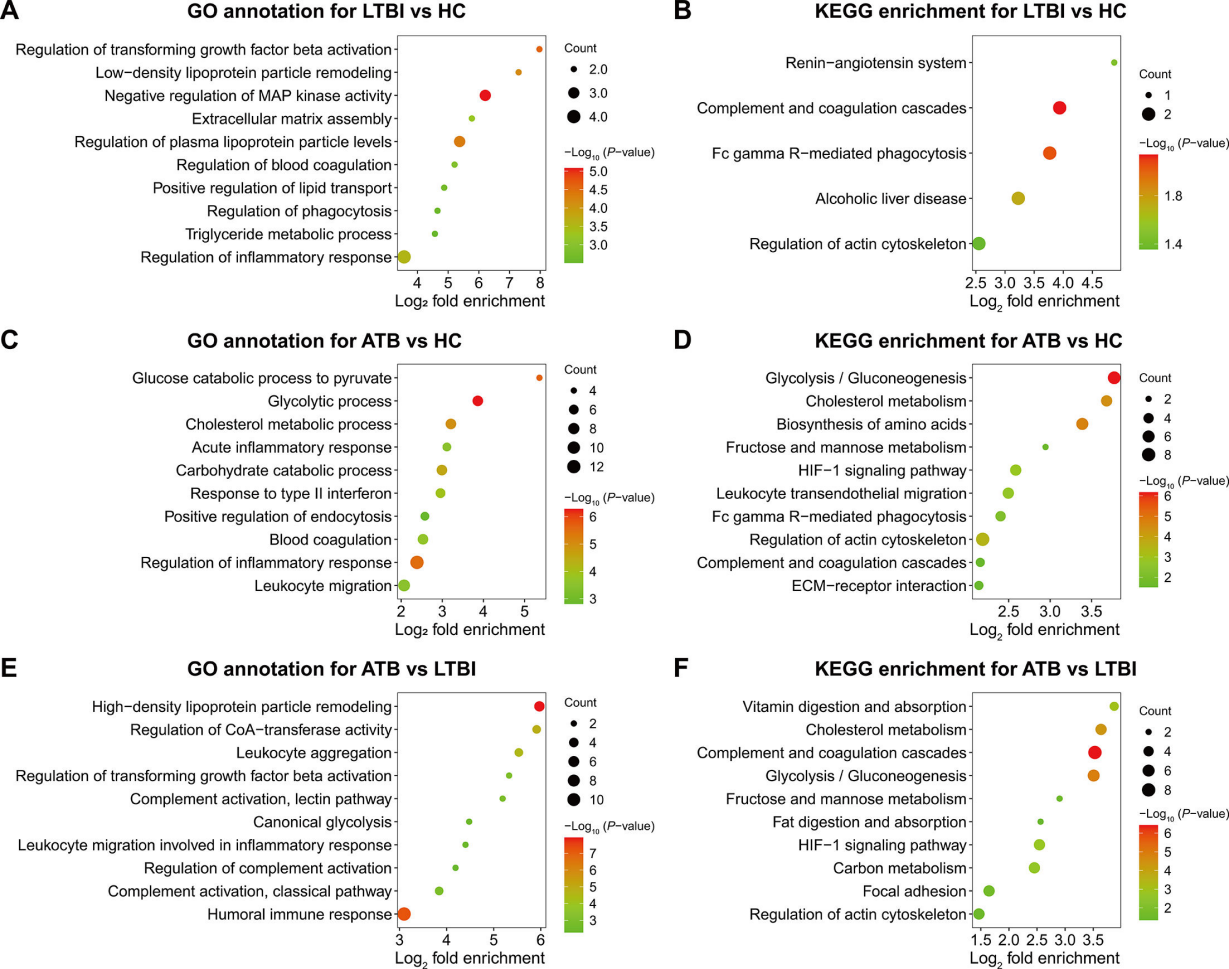
GO annotation and KEGG pathway analyses of DEPs between different TB stages. (**A**) The bubble plot shows significantly enriched BPs for DEPs between LTBI and HC. (**B**) The bubble plot shows significantly enriched KEGG pathways for DEPs between LTBI and HC. (**C**) The bubble plot shows significantly enriched BPs for DEPs between ATB and HC. (**D**) The bubble plot shows significantly enriched KEGG pathways for DEPs between ATB and HC. (**E**) The bubble plot shows significantly enriched BPs for DEPs between ATB and LTBI. (**F**) The bubble plot shows significantly enriched KEGG pathways for DEPs between ATB and LTBI. Dot size represents the number of DEPs, and the color of dots denotes the enrichment significance.

As for the DEPs between LTBI and HC, GO analyses showed that significant biological processes were enriched in negative regulation of mitogen-activated protein (MAP) kinase activity, regulation of plasma lipoprotein particle levels, regulation of inflammatory response, and regulation of blood coagulation (Fig. 2A and Table S4). The enriched KEGG pathway included complement and coagulation cascades and Fc gamma R-mediated phagocytosis (Fig. 2B and Table S5). In terms of DEPs between ATB and HC, proteins were more prone to be involved in glycolytic process, cholesterol metabolic process, regulation of inflammatory response, and blood coagulation (Fig. 2C and Table S4). Furthermore, KEGG analyses also found enriched pathways in glycolysis/gluconeogenesis, cholesterol metabolism, HIF-1 signaling pathway, Fc gamma R-mediated phagocytosis, and complement and coagulation cascades (Fig. 2D and Table S5). After the enrichment analysis of GO terms, the DEPs between ATB and LTBI were primarily enriched in high-density lipoprotein particle remodeling, humoral immune response, and leukocyte aggregation. KEGG analysis detected enriched pathways similar to those found by enrichment analysis on the DEPs between ATB and HC (Fig. 2E and F and Table S4 and S5).

### PPI network of DEPs and hub protein analyses

**Fig 3:**
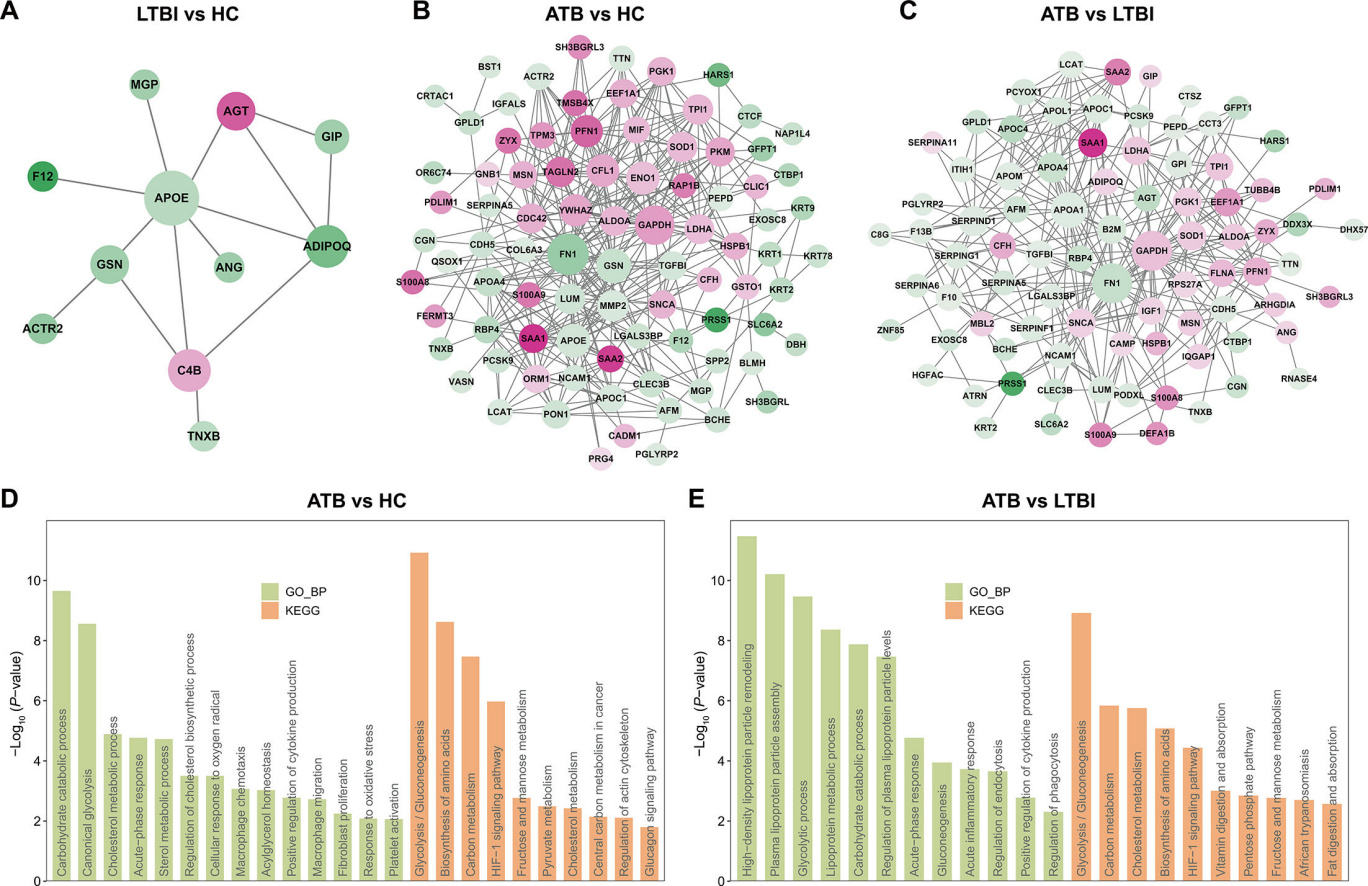
PPI analyses on the DEPs between groups. Node size denotes the centrality degree of proteins. Green indicates lower log-transformed FC of protein abundance, and red indicates higher log-transformed FC of protein abundance. (**A**) PPI network diagram shows the interaction among proteins differentially expressed between LTBI and HC. (**B**) PPI network diagram shows the interaction among proteins differentially expressed between ATB and HC. (**C**) PPI network diagram shows the interaction among proteins differentially expressed between ATB and LTBI. (**D**) GO and KEGG analyses on core DEPs between ATB and HC using MCC method. (**E**) GO and KEGG analyses on core DEPs between ATB and LTBI using MCC method.

This study constructed PPI networks of DEPs using STRING database, which has a collection of known and predicted protein-protein interactions. The PPI network for DEPs between LTBI and HC consisted of 11 nodes and 14 edges, with a PPI enrichment *P*-value of 1.39e-07 (Fig. 3A and Table S6). More nodes and edges were observed in the network for DEPs between ATB and HC, the counts of which were 87 and 368, respectively (Fig. 3B and Table S6). Similarly, there were 85 nodes and 333 edges in the PPI network for DEPs between ATB and LTBI (Fig. 3C and Table S6).

Additionally, we utilized the MCC method to evaluate the centrality of plasma DEPs, of which the top 20 were further subjected to perform GO and KEGG enrichment analyses (Table S6). For the core DEPs between ATB and HC, GO annotation found significant enrichment in carbohydrate catabolic process, cholesterol metabolic process, acute-phase response, macrophage chemotaxis, positive regulation of cytokine production, and platelet activation (Fig. 3D). KEGG analyses also indicated enrichment in glycolysis/gluconeogenesis, HIF-1 signaling pathway, and cholesterol metabolism (Fig. 3D). Similar enrichment patterns were also found for the core DEPs between ATB and LTBI (Fig. 3E).

### *Mtb* infection triggered co-expression of proteome modules

**Fig 4:**
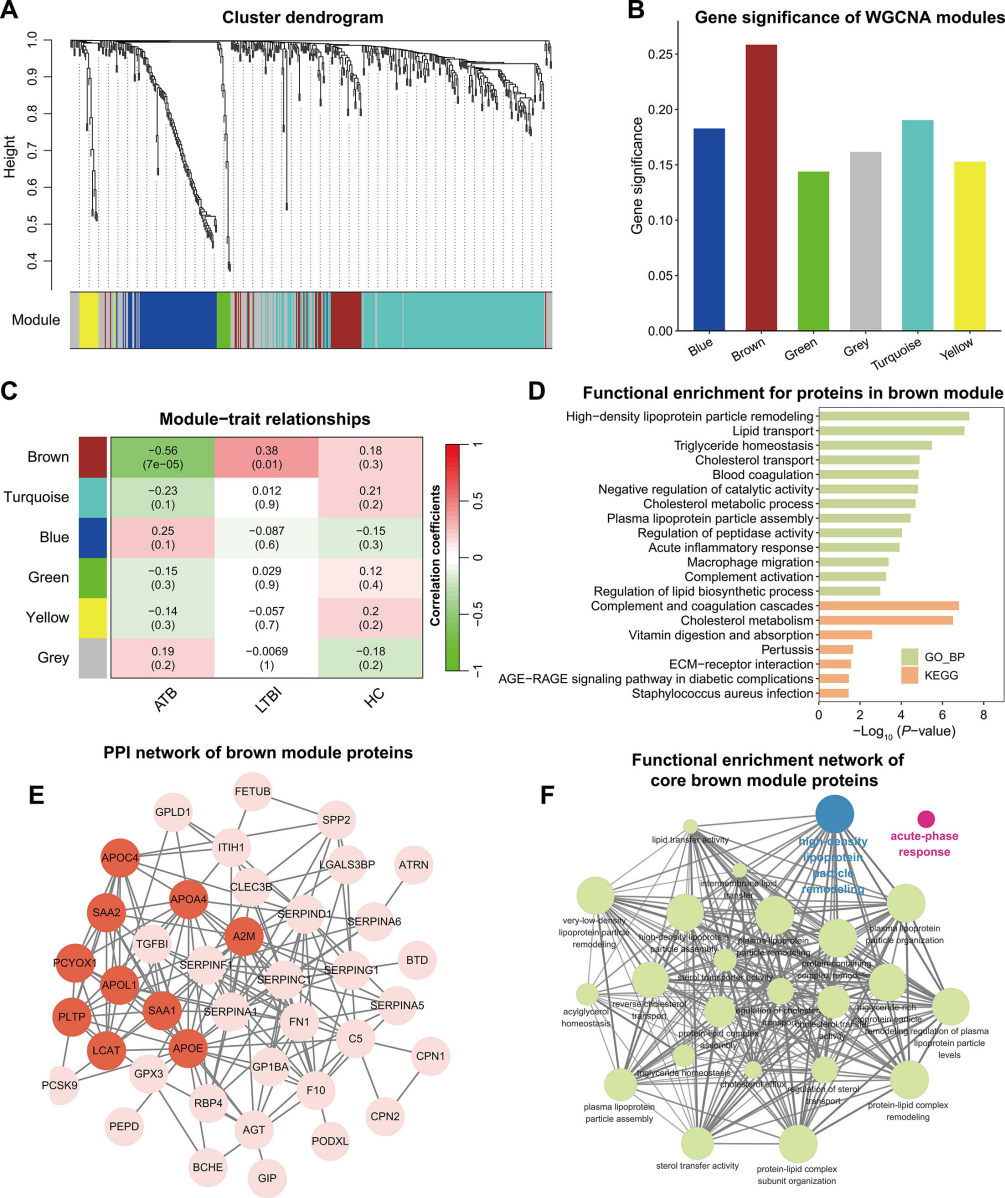
WGCNA analyses based on plasma proteomic data from 45 participants. (**A**) Protein modules generated using hierarchical clustering. (**B**) Bar plot presents the average gene significance in each module associated with TB stages. Bars are colored based on module names. (**C**) Heatmap shows the correlation between module eigenvalues and disease stages. The numbers preceding the parentheses denote correlation coefficients, and those inside the parentheses are *P*-values. Red represents a positive relationship, and green represents a negative relationship. (**D**) GO enrichment and KEGG pathway analyses on proteins in brown modules. (**E**) PPI network among proteins in brown module. Nodes in red denote core proteins identified by the MCC method. (**F**) Functional enrichment network from ClueGo for core proteins in the brown module. Node represents signaling pathways, with a larger node size indicating a higher enrichment significance. Edge represents the correlation between functions, with a thicker edge indicating a greater kappa coefficient between functions. The most significant pathway involving lipid metabolism was colored in blue, and the most significant pathway involving acute-phase response was colored in red.

Before formal WGCNA analyses, one sample was removed to avoid inducing potential biases given its distinct clustering location (Fig. S3A). Then, we calculated the correlation coefficients for protein pairs, which were further weighted (power = 5) to perform hierarchical clustering, thereby grouping proteins into various modules (Fig. S3B). In this study, WGCNA revealed six modules, of which the turquoise module encompassed the largest number of proteins (203 proteins), followed by the blue module (84 proteins) and the brown module (50 proteins; Fig. 4A and Table S7). Analyses on the association of proteins with disease stages found a higher level of gene significance in the brown module (*r*_mean_ = 0.26) and turquoise module (*r*_mean_ = 0.19; Fig. 4B). Intriguingly, module-trait relationship analyses suggested that brown module was positively upregulated in LTBI (*r* = 0.38, *P* = 0.01), while the association was reversed for those with ATB (*r* = −0.56, *P* < 0.001). Similar findings were shown in the relationship of disease conditions with turquoise, green, and yellow modules, albeit with no statistical significance (Fig. 4C).

Here, the brown module was further scrutinized to gain insight into the function involved in TB disease. With regard to GO annotation, results showed that the proteins were involved in high-density lipoprotein particle remodeling (*P* = 5.03e−08), transport of lipid (*P* = 8.66e−08) and cholesterol (*P* = 1.31e−05), blood coagulation (*P* = 1.45e−05), cholesterol metabolic process (*P* = 2.01e−05), acute inflammatory response (*P* = 1.22e−04), and complement activation (*P* = 5.46e−04; Fig. 4D). Similarly, KEGG pathway found proteins in the brown module were significantly enriched in complement and coagulation cascades (*P* = 1.59e−07), cholesterol metabolism (*P* = 3.11e−07), and extracellular matrix (ECM)-receptor interaction (*P* = 2.77E−02; Fig. 4D). Additionally, we implemented PPI analyses for proteins in brown modules based on STRING database with default parameters, and the results suggested significant PPI enrichment (*P* < 1e−16), with a node degree of 6.73 on average (Fig. 4E). The top 10 proteins ranked by the MCC algorithm were viewed as core proteins, including APOE, APOA4, APOL1, SAA1, SAA2, LCAT, PLTP, APOC4, PCYOX1, and A2M (Fig. 4E). Furthermore, we performed ClueGO enrichment analysis and found those proteins were mostly enriched in two domains, lipid metabolism pathways and acute-phase response (Fig. 4F).

### Validation and evaluation of key proteins in differentiating TB conditions

**Fig 5:**
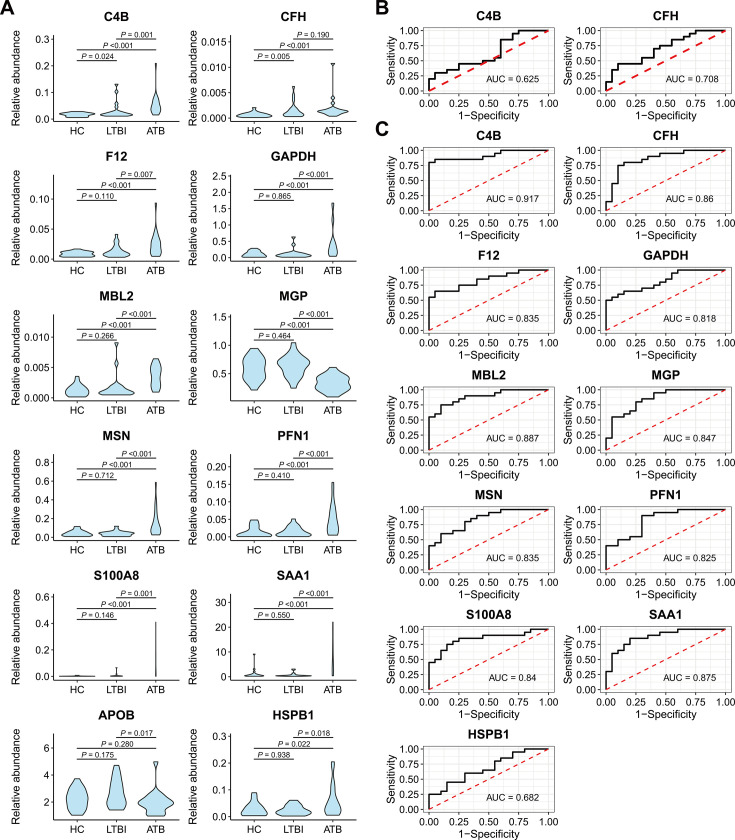
Protein abundance and diagnostic efficiency of DEPs in the validation cohort. (**A**) Violin diagram presents the abundance distribution of DEPs using the PRM method. Between-group comparisons are implemented using DEP2 packages in R software. (**B**) ROC curves of two DEPs in differentiating LTBI and HC. (**C**) ROC curves of 11 DEPs in differentiating ATB and HC.

Considering the above findings over differential expression level, functional enrichment, and protein centrality through PPI analyses, we quantified 19 proteins to validate corresponding proteomic profiles using the PRM method among 60 samples (Table S8). Of 19 proteins, 12 were found to be significantly different in at least one group pair (Table 1 and Fig. 5A). Specifically, we detected 2 upregulated proteins (C4B and CFH) in LTBI, 10 upregulated proteins (C4B, CFH, F12, GAPDH, MBL2, MSN, PFN1, S100A8, SAA1, and HSPB1), and 1 downregulated protein (matrix Gla protein [MGP]) in ATB, compared with HC (Table 1 and Fig. 5A). In addition, the abundance of APOB decreased when comparing ATB and LTBI, but not in the aforementioned group pairs (Table 1 and Fig. 5A).

**TABLE 1:** Plasma DEPs identified by PRM^*a*^

Protein	Abundance			LTBI vs HC		ATB vs HC		ATB vs LTBI	
	HC	LTBI	ATB	LogFC	*P*-value	LogFC	*P*-value	LogFC	*P*-value
C4B	0.018	0.019	0.043	0.64	0.024	1.57	<0.001	0.93	0.001
CFH	5.34E−04	7.66E−04	0.001	1.04	0.005	1.52	<0.001	0.47	0.190
F12	0.008	0.009	0.024	0.49	0.110	1.33	<0.001	0.84	0.007
GAPDH	0.102	0.072	0.273	0.08	0.865	2.01	<0.001	1.93	<0.001
MBL2	0.001	0.001	0.004	0.39	0.266	1.81	<0.001	1.42	<0.001
MGP	0.604	0.667	0.302	0.14	0.464	−0.87	<0.001	−1.01	<0.001
MSN	0.027	0.029	0.071	−0.13	0.712	1.68	<0.001	1.81	<0.001
PFN1	0.007	0.009	0.035	0.44	0.410	2.31	<0.001	1.88	<0.001
S100A8	0.001	0.003	0.004	0.91	0.146	2.97	<0.001	2.07	0.001
SAA1	0.506	0.420	3.672	−0.27	0.550	2.48	<0.001	2.75	<0.001
APOB	2.275	2.362	1.872	0.25	0.175	−0.2	0.280	−0.44	0.017
HSPB1	0.023	0.020	0.034	−0.04	0.938	1.06	0.022	1.10	0.018

^*a*^ C4B denotes complement C4-B. F12 denotes coagulation factor XII. GAPDH denotes glyceraldehyde-3-phosphate dehydrogenase. MBL2 denotes mannose-binding protein C. MSN denotes moesin. PFN1 denotes profilin-1. S100A8 denotes protein S100-A8. SAA1 denotes serum amyloid A-1 protein. APOB denotes apolipoprotein B. HSPB1 denotes heat shock protein family B member 1.

Furthermore, we assessed the efficacy of DEPs in distinguishing different TB stages. Compared with C4B, CFH had a better performance in differentiating LTBI and HC, with an overall AUC value of 0.708 (95% CI, 0.546–0.869; Fig. 5B and Table S9). Interestingly, when differentiating ATB and HC, C4B achieves a maximum AUC value up to 0.917 (95% CI, 0.823–1), followed by MBL2 (AUC, 0.887; 95% CI, 0.788–0.987) and SAA1 (AUC, 0.875; 95% CI, 0.767–0.982; Fig. 5C and Table S9). Moreover, SAA1 (AUC, 0.917; 95% CI, 0.836–0.999) and MGP (AUC, 0.905; 95% CI, 0.809–1.000) outweighed other proteins in discriminating ATB from LTBI (Fig. S4 and Table S9).

## DISCUSSION

In this study, we employed MS-based proteomics technology to demonstrate the differential plasma proteome profiling across TB stages, and several potential biomarkers were found to be valuable in discriminating clinical conditions following *Mtb* infection. PRM protein quantification revealed that CFH had a better performance in differentiating LTBI and HC than C4B, while C4B, MBL2, and SAA1 favored the discrimination of ATB from HC, and SAA1 and MGP suggested potential values in differentiating ATB from LTBI.

Additionally, GO and KEGG enrichment analyses confirmed that TB stages correlated with functional changes in lipid metabolism and carbohydrate catabolism. Notably, the findings of functional enrichment analyses literally suggested the presence of abnormal energy metabolism along with *Mtb* infection, while whether such alterations were attributable to disease development requires further functional validation. As a wasting disease, TB usually triggers malnutrition, metabolic disorders, fever, and weight loss (42). Nutrients from hosts, like carbon and nitrogen, were employed to promote bacterial growth, and fatty acids are found to be the major source of energy for *Mtb* during infection (43, 44). Marrero et al. (45) found that the depletion of phosphoenolpyruvate carboxykinase contributed to mycobacterial clearance in mice, indicating that the gluconeogenesis pathway is critical for *Mtb* to maintain infection. Besides, prior evidence showed that proteins associated with immunological processes, such as complement cascade activation, were elevated at early stages of TB progression and were regarded as biomarkers in discerning high-risk populations, which is consistent with our study (46). Similarly, Mateos et al. (47) reported that proteome profiles of serum from ATB patients were featured by elevated proteins involved in complement activation, inflammation, and regulation of immune response, as well as the decrease of apolipoprotein A and serotransferrin, further demonstrating the importance of lipid transport in TB pathogenesis.

To identify potential biomarkers in differentiating different TB stages, this study employed PRM to further determine the abundance of hub proteins using plasma from the validation cohort. Compared to HC, we found elevated expression levels of CFH in LTBI, and C4B, MBL2, and SAA1 in ATB, and those proteins showed good diagnostic value in differentiating disease conditions following *Mtb* infection. The complement system is an important component of the innate immune, capable of unleashing robust cytotoxic and inflammatory mechanisms upon activation. CFH functions as a potent inhibitor within the complement cascade, exerting a negative regulatory role in the activation of complement C3 (48). Microorganisms have evolved diverse strategies to circumvent host immune responses, among which the hijacking of CFH from the host represents a mechanism employed by pathogens to evade complement-mediated attack (49–52). In brief, pathogens could bind CFH by adapting their surfaces, like the expression of molecular markers of host cells or specific CFH-binding proteins (53). After binding to the pathogen surface, CFH maintains its role in negatively regulating the activation of complement proteins surrounding the pathogens (53). Several pathogens cleave CFH by secreting specific proteases, and the inactivation of CFH leads to unchecked complement activation, which further contributes to the depletion of complement proteins (54, 55). Also, CFH may act as a bridging molecule between complement receptor 3 and pathogens to promote adhesion, facilitating pathogen entry into host cells (56, 57). Moreover, bacteria-bound CFH is reported to compete with C1q for binding, thereby inhibiting the initiation of the classical complement pathway (58). In populations infected with *Mtb*, especially for ATB, we have observed differential elevations in the plasma complement proteins CFH, implicating the possibility that *Mtb* may evade the host immune response through similar mechanisms. Previous research has also revealed that CFHR2 and CFHR3 exhibit favorable diagnostic performance in osteoarticular TB, suggesting that CFH and its related proteins play a significant role in host immune response against *Mtb* (59). Furthermore, Chu et al. (60) concluded that higher levels of complement C4 were positively associated with the presence of cavities in male TB patients, supporting the findings in our work.

Mannose-binding lectin (MBL), a pivotal component in innate immunity, can facilitate phagocytosis and the activation of the lectin complement pathway (61, 62). A prior report has demonstrated a significant correlation between MBL2 gene polymorphisms and susceptibility to TB among the Chinese population (63). Our study revealed that plasma MBL2 concentration was markedly elevated in ATB, in comparison with HC. Besides, we found that patients with ATB were likely to experience a substantial elevation of plasma SAA1 level. Kawka et al. (64) proposed that *Mtb* can bind to human SAA1, thereby facilitating its internalization into macrophages and enhancing the intracellular growth and survival of the bacterium. Moreover, the binding between *Mtb* and SAA1 has been shown to modulate the transcriptional response of the pathogen, resulting in the upregulation of genes encoding virulence-associated proteins (64). Previous quantitative proteomics demonstrated a significant increase of serum SAA1 in ATB patients compared to the HC group (65), and this correlation was also validated by another three studies (22, 25, 66). Similarly, a study of patients undergoing anti-TB treatment showed, compared to the baseline, a significant reduction in serum SAA1 level after the intensive phase of therapy (the first 2 months after treatment initiation), suggesting that SAA1 may also serve as a potential biomarker for monitoring treatment efficacy (67). Additionally, ATB patients in our study showed a significant reduction in plasma MGP level. To our knowledge, our study is the first to report MGP as a potential biomarker of ATB. As a natural calcification inhibitor, MGP plays an essential role in regulating cellular calcium concentration (68). An animal experiment conducted by Li et al. (69) suggested decreased MGP expression in rats with artery calcification when compared to the control group. Another study targeting MGP^−/−^ mice highlighted the inhibitory effects on calcification in the arterial system, and the reestablishment of MGP expression can alleviate arterial calcification (70, 71). Consistently, prior evidence supports that MGP is associated with the incidence of kidney stone and renal calcification (72). Based on existing findings, we hypothesize that the change in plasma MGP abundance might result from host protective responses to *Mtb* infection, since decreased MGP expression promotes the formation of calcified lesions, thereby enabling hosts to reduce the risk of *Mtb* dissemination. However, the exact pathological mechanisms of MGP underlying TB development are limited and poorly understood, and more studies are necessary to uncover the pathways behind the observed phenomenon.

By far, other proteomic biomarkers have been discovered with diagnostic potential in discerning TB stages. A label-free quantitative proteomics identified 31 overlapping proteins with significant difference in expression level among pulmonary TB patients, and a diagnostic model consisting of ACT, AGP1, and CDH1 was established and presented a sensitivity of 82.3% and a specificity of 95.2% in discriminating pulmonary TB from LTBI and a sensitivity of 81.2% and a specificity of 90.1% in discriminating pulmonary TB from HC (73). Our study has further confirmed the potential protein biomarkers from plasma that can distinguish between different disease states of TB, among which C4B, MBL2, and SAA1 are helpful in the discrimination of ATB from HC, CFH in the discrimination of LTBI from HC, and SAA1 and MGP in the discrimination of ATB from LTBI. Similarly, Franco Fontes et al. (66) reported that SAA, HDL-C, and APOA1 were suitable as triage signatures for ATB, aligning with our findings. A nested case-control study conducted in Malawi and South Africa found that CFH and eight other host proteins constituted a promising classifier for TB identification, which echoes the upregulated trend of CFH among LTBI and ATB in the present work (26). In line with our research, Ahmadi et al. (74) revealed a high level of serum MBL concentration in TB patients with non-TB individuals as the control group, and comparable results were also reported by García-Gasalla et al. (75) and Selvaraj et al. (76). To date, the role of MGP as a protein signature of TB patients has not been reported in the literature, and this study extends MGP as a new biomarker for such signatures. Notably, it is acknowledged that existing research on proteomic biomarkers associated with distinct disease conditions presents substantial heterogeneity, and there needs to be more efforts to further validate the plasma protein biomarkers that have hitherto been identified.

Compared to transcriptome biomarkers, which have great potential but are difficult to apply in portable devices due to the complex isolation process and unstable molecular structure, streamlined proteomic signatures are more suitable for low-cost and large-scale detection. Nevertheless, our study has several limitations. First, although this study has suggested the differential proteome profiles and potential protein biomarkers in discriminating disease stages following *Mtb* infection, the results are partially limited by a relatively small sample size, and larger cohorts are needed to further validate our findings. Second, it is necessary to establish more follow-up cohorts to track the onset of ATB among LTBI, which could be helpful in revealing prognostic biomarkers for TB. Third, since this study just identified CFH as a potential signature for the discrimination between LTBI and HC, there exists a risk of misdiagnosis if relying on a single biomarker, and more markers are warranted to obtain a higher diagnostic accuracy. Additionally, ATB patients in this study are only the individuals with pulmonary TB; there requires further work to capture the proteomic profiling and protein biomarkers among those with extrapulmonary TB.

In conclusion, our analyses illustrate the hub plasma proteins correlated with disease stages following *Mtb* infection and provide the potential diagnostic biomarkers for an accurate diagnosis of the infection stages. Our research also confirms that carbohydrate catabolism, metabolism of cholesterol and lipid, immune response and inflammation, complement and coagulation cascades are associated with TB pathogenesis, offering insights into the mechanisms explaining the distinct outcomes following *Mtb* infection.

## Data Availability

The clean proteome data have been uploaded to a public GitHub depository at https://github.com/an94maley/Proteomics_TB.

## SUPPLEMENTAL MATERIAL

Supplemental Figures msystems.00616-25-s0001.docx Figures S1 to S4.

Supplemental Tables msystems.00616-25-s0002.xlsx Tables S1 to S9.

ASM does not own the copyrights to Supplemental Material that may be linked to, or accessed through, an article. The authors have granted ASM a non-exclusive, world-wide license to publish the Supplemental Material files. Please contact the corresponding author directly for reuse.

## References

[1] WHO. 2024. Globel tuberculosis report 2024. Available from: https://www.who.int/teams/global-tuberculosis-programme/tb-reports/global-tuberculosis-report-2024

[2] Richeldi L. 2006. An update on the diagnosis of tuberculosis infection. Am J Respir Crit Care Med 174:736–742. doi:10.1164/rccm.200509-1516PP 16799073

[3] Getahun H, Matteelli A, Chaisson RE, Raviglione M. 2015. Latent Mycobacterium tuberculosis infection. N Engl J Med 372:2127–2135. doi:10.1056/NEJMra1405427 26017823

[4] Vynnycky E, Fine PE. 2000. Lifetime risks, incubation period, and serial interval of tuberculosis. Am J Epidemiol 152:247–263. doi:10.1093/aje/152.3.247 10933272

[5] Behr MA, Edelstein PH, Ramakrishnan L. 2018. Revisiting the timetable of tuberculosis. BMJ 362:k2738. doi:10.1136/bmj.k2738 30139910 PMC6105930

[6] Behr MA, Edelstein PH, Ramakrishnan L. 2019. Is Mycobacterium tuberculosis infection life long. BMJ 367:l5770. doi:10.1136/bmj.l5770 31649096 PMC6812595

[7] Behr MA, Kaufmann E, Duffin J, Edelstein PH, Ramakrishnan L. 2021. Latent tuberculosis: two centuries of confusion. Am J Respir Crit Care Med 204:142–148. doi:10.1164/rccm.202011-4239PP 33761302 PMC8650795

[8] Emery JC, Richards AS, Dale KD, McQuaid CF, White RG, Denholm JT, Houben RMGJ. 2021. Self-clearance of Mycobacterium tuberculosis infection: implications for lifetime risk and population at-risk of tuberculosis disease. Proc Biol Sci 288:20201635. doi:10.1098/rspb.2020.1635 33467995 PMC7893269

[9] Swindells S, Ramchandani R, Gupta A, Benson CA, Leon Cruz J, Mwelase N, Jean Juste MA, Lama JR, Valencia J, Omoz-Oarhe A, Supparatpinyo K, Masheto G, Mohapi L. 2019. One month of rifapentine plus isoniazid to prevent HIV-related Tuberculosis. N Engl J Med 380:1001–1011. doi:10.1056/NEJMoa1806808 30865794 PMC6563914

[10] Sterling TR, Villarino ME, Borisov AS, Shang N, Gordin F, Bliven-Sizemore E, Hackman J, Hamilton CD, Menzies D, Kerrigan A, Weis SE, Weiner M, Wing D, Conde MB, Bozeman L, Horsburgh CR, Chaisson RE, Team T. 2011. Three months of rifapentine and isoniazid for latent tuberculosis infection. N Engl J Med 365:2155–2166. doi:10.1056/NEJMoa1104875 22150035

[11] Rangaka MX, Wilkinson KA, Glynn JR, Ling D, Menzies D, Mwansa-Kambafwile J, Fielding K, Wilkinson RJ, Pai M. 2012. Predictive value of interferon-γ release assays for incident active tuberculosis: a systematic review and meta-analysis. Lancet Infect Dis 12:45–55. doi:10.1016/S1473-3099(11)70210-9 21846592 PMC3568693

[12] Pai M, Denkinger CM, Kik SV, Rangaka MX, Zwerling A, Oxlade O, Metcalfe JZ, Cattamanchi A, Dowdy DW, Dheda K, Banaei N. 2014. Gamma interferon release assays for detection of Mycobacterium tuberculosis infection. Clin Microbiol Rev 27:3–20. doi:10.1128/CMR.00034-13 24396134 PMC3910908

[13] Lalvani A, Berrocal-Almanza LC, Halliday A. 2019. Predicting progression to active tuberculosis: a rate-limiting step on the path to elimination. PLoS Med 16:e1002814. doi:10.1371/journal.pmed.1002814 31125334 PMC6534286

[14] Huang Y, Ai L, Wang X, Sun Z, Wang F. 2022. Review and updates on the diagnosis of tuberculosis. J Clin Med 11:5826. doi:10.3390/jcm11195826 36233689 PMC9570811

[15] Tabone O, Verma R, Singhania A, Chakravarty P, Branchett WJ, Graham CM, Lee J, Trang T, Reynier F, Leissner P, Kaiser K, Rodrigue M, Woltmann G, Haldar P, O’Garra A. 2021. Blood transcriptomics reveal the evolution and resolution of the immune response in tuberculosis. J Exp Med 218:e20210915. doi:10.1084/jem.20210915 34491266 PMC8493863

[16] Zak DE, Penn-Nicholson A, Scriba TJ, Thompson E, Suliman S, Amon LM, Mahomed H, Erasmus M, Whatney W, Hussey GD, et al.. 2016. A blood RNA signature for tuberculosis disease risk: a prospective cohort study. Lancet 387:2312–2322. doi:10.1016/S0140-6736(15)01316-1 27017310 PMC5392204

[17] Suliman S, Thompson EG, Sutherland J, Weiner J 3rd, Ota MOC, Shankar S, Penn-Nicholson A, Thiel B, Erasmus M, Maertzdorf J, et al.. 2018. Four-gene pan-African blood signature predicts progression to tuberculosis. Am J Respir Crit Care Med 197:1198–1208. doi:10.1164/rccm.201711-2340OC 29624071 PMC6019933

[18] Schiff HF, Walker NF, Ugarte-Gil C, Tebruegge M, Manousopoulou A, Garbis SD, Mansour S, Wong PHM, Rockett G, Piazza P, Niranjan M, Vallejo AF, Woelk CH, Wilkinson RJ, Tezera LB, Garay-Baquero D, Elkington P. 2024. Integrated plasma proteomics identifies tuberculosis-specific diagnostic biomarkers. JCI Insight 9:e173273. doi:10.1172/jci.insight.173273 38512356 PMC11141874

[19] Yoon C, Chaisson LH, Patel SM, Allen IE, Drain PK, Wilson D, Cattamanchi A. 2017. Diagnostic accuracy of C-reactive protein for active pulmonary tuberculosis: a meta-analysis. Int J Tuberc Lung Dis 21:1013–1019. doi:10.5588/ijtld.17.0078 28826451 PMC5633000

[20] Garlant HN, Ellappan K, Hewitt M, Perumal P, Pekeleke S, Wand N, Southern J, Kumar SV, Belgode H, Abubakar I, Sinha S, Vasan S, Joseph NM, Kempsell KE. 2022. Evaluation of host protein biomarkers by ELISA from whole lysed peripheral blood for development of diagnostic tests for active tuberculosis. Front Immunol 13:854327. doi:10.3389/fimmu.2022.854327 35720382 PMC9205408

[21] Kumar NP, Banurekha VV, Nair D, Dolla C, Kumaran P, Babu S. 2018. Modulation of iron status biomarkers in tuberculosis-diabetes co-morbidity. Tuberculosis (Edinburgh 108:127–135. doi:10.1016/j.tube.2017.11.011 PMC5846115 29523313

[22] Andrade BB, Pavan Kumar N, Mayer-Barber KD, Barber DL, Sridhar R, Rekha VVB, Jawahar MS, Nutman TB, Sher A, Babu S. 2013. Plasma heme oxygenase-1 levels distinguish latent or successfully treated human tuberculosis from active disease. PLoS One 8:e62618. doi:10.1371/journal.pone.0062618 23671613 PMC3646008

[23] Kathamuthu GR, Kumar NP, Moideen K, Nair D, Banurekha VV, Sridhar R, Baskaran D, Babu S. 2020. Matrix metalloproteinases and tissue inhibitors of metalloproteinases are potential biomarkers of pulmonary and extra-pulmonary tuberculosis. Front Immunol 11:419. doi:10.3389/fimmu.2020.00419 32218787 PMC7078103

[24] Chegou NN, Sutherland JS, Malherbe S, Crampin AC, Corstjens PLAM, Geluk A, Mayanja-Kizza H, Loxton AG, van der Spuy G, Stanley K, Kotzé LA, van der Vyver M, Rosenkrands I, Kidd M, van Helden PD, Dockrell HM, Ottenhoff THM, Kaufmann SHE, Walzl G. 2016. Diagnostic performance of a seven-marker serum protein biosignature for the diagnosis of active TB disease in African primary healthcare clinic attendees with signs and symptoms suggestive of TB. Thorax 71:785–794. doi:10.1136/thoraxjnl-2015-207999 27146200

[25] De Groote MA, Sterling DG, Hraha T, Russell TM, Green LS, Wall K, Kraemer S, Ostroff R, Janjic N, Ochsner UA. 2017. Discovery and validation of a six-marker serum protein signature for the diagnosis of active pulmonary tuberculosis. J Clin Microbiol 55:3057–3071. doi:10.1128/JCM.00467-17 28794177 PMC5625392

[26] Morris TC, Hoggart CJ, Chegou NN, Kidd M, Oni T, Goliath R, Wilkinson KA, Dockrell HM, Sichali L, Banda L, Crampin AC, French N, Walzl G, Levin M, Wilkinson RJ, Hamilton MS. 2021. Evaluation of host serum protein biomarkers of tuberculosis in sub-Saharan Africa. Front Immunol 12:639174. doi:10.3389/fimmu.2021.639174 33717190 PMC7947659

[27] Sester M, Sotgiu G, Lange C, Giehl C, Girardi E, Migliori GB, Bossink A, Dheda K, Diel R, Dominguez J, Lipman M, Nemeth J, Ravn P, Winkler S, Huitric E, Sandgren A, Manissero D. 2011. Interferon-γ release assays for the diagnosis of active tuberculosis: a systematic review and meta-analysis. Eur Respir J 37:100–111. doi:10.1183/09031936.00114810 20847080

[28] Pai M, Zwerling A, Menzies D. 2008. Systematic review: T-cell-based assays for the diagnosis of latent tuberculosis infection: an update. Ann Intern Med 149:177–184. doi:10.7326/0003-4819-149-3-200808050-00241 18593687 PMC2951987

[29] Ayers T, Hill AN, Raykin J, Mohanty S, Belknap RW, Brostrom R, Khurana R, Lauzardo M, Miller TL, Narita M, Pettit AC, Pyan A, Salcedo KL, Polony A, Flood J. 2024. Comparison of tuberculin skin testing and interferon-γ release assays in predicting tuberculosis disease. JAMA Netw Open 7:e244769. doi:10.1001/jamanetworkopen.2024.4769 38568690 PMC10993073

[30] Chen S, Siedhoff HR, Zhang H, Liu P, Balderrama A, Li R, Johnson C, Greenlief CM, Koopmans B, Hoffman T, DePalma RG, Li D-P, Cui J, Gu Z. 2022. Low-intensity blast induces acute glutamatergic hyperexcitability in mouse hippocampus leading to long-term learning deficits and altered expression of proteins involved in synaptic plasticity and serine protease inhibitors. Neurobiol Dis 165:105634. doi:10.1016/j.nbd.2022.105634 35077822 PMC12928849

[31] Mun DG, Vanderboom PM, Madugundu AK, Garapati K, Chavan S, Peterson JA, Saraswat M, Pandey A. 2021. DIA-based proteome profiling of nasopharyngeal swabs from COVID-19 patients. J Proteome Res 20:4165–4175. doi:10.1021/acs.jproteome.1c00506 34292740

[32] Consortium U. 2023. UniProt: the universal protein knowledgebase in 2023. Nucleic Acids Res 51:D523–D531. doi:10.1093/nar/gkac1052 36408920 PMC9825514

[33] Feng Z, Fang P, Zheng H, Zhang X. 2023. DEP2: an upgraded comprehensive analysis toolkit for quantitative proteomics data. Bioinformatics 39:btad526. doi:10.1093/bioinformatics/btad526 37624922 PMC10466079

[34] Szklarczyk D, Kirsch R, Koutrouli M, Nastou K, Mehryary F, Hachilif R, Gable AL, Fang T, Doncheva NT, Pyysalo S, Bork P, Jensen LJ, von Mering C. 2023. The STRING database in 2023: protein-protein association networks and functional enrichment analyses for any sequenced genome of interest. Nucleic Acids Res 51:D638–D646. doi:10.1093/nar/gkac1000 36370105 PMC9825434

[35] Chin CH, Chen SH, Wu HH, Ho CW, Ko MT, Lin CY. 2014. cytoHubba: identifying hub objects and sub-networks from complex interactome. BMC Syst Biol 8 Suppl 4:S11. doi:10.1186/1752-0509-8-S4-S11 25521941 PMC4290687

[36] Langfelder P, Horvath S. 2008. WGCNA: an R package for weighted correlation network analysis. BMC Bioinformatics 9:559. doi:10.1186/1471-2105-9-559 19114008 PMC2631488

[37] Karpievitch YV, Dabney AR, Smith RD. 2012. Normalization and missing value imputation for label-free LC-MS analysis. BMC Bioinformatics 13 Suppl 16:S5. doi:10.1186/1471-2105-13-S16-S5 PMC3489534 23176322

[38] Hrydziuszko O, Viant MR. 2012. Missing values in mass spectrometry based metabolomics: an undervalued step in the data processing pipeline. Metabolomics (Los Angeles) 8:161–174. doi:10.1007/s11306-011-0366-4

[39] Marhaug G, Dowton SB. 1994. Serum amyloid A: an acute phase apolipoprotein and precursor of AA amyloid. Baillieres Clin Rheumatol 8:553–573. doi:10.1016/s0950-3579(05)80115-3 7525085

[40] Song C, Hsu K, Yamen E, Yan W, Fock J, Witting PK, Geczy CL, Freedman SB. 2009. Serum amyloid A induction of cytokines in monocytes/macrophages and lymphocytes. Atherosclerosis 207:374–383. doi:10.1016/j.atherosclerosis.2009.05.007 19535079

[41] Wu D, Zhang M, Xu J, Song E, Lv Y, Tang S, Zhang X, Kemper N, Hartung J, Bao E. 2016. In vitro evaluation of aspirin-induced HspB1 against heat stress damage in chicken myocardial cells. Cell Stress Chaperones 21:405–413. doi:10.1007/s12192-016-0666-8 26910344 PMC4837179

[42] Hood MLH. 2013. A narrative review of recent progress in understanding the relationship between tuberculosis and protein energy malnutrition. Eur J Clin Nutr 67:1122–1128. doi:10.1038/ejcn.2013.143 23942176

[43] Ehrt S, Schnappinger D, Rhee KY. 2018. Metabolic principles of persistence and pathogenicity in Mycobacterium tuberculosis. Nat Rev Microbiol 16:496–507. doi:10.1038/s41579-018-0013-4 29691481 PMC6045436

[44] Kondo E, Kanai K. 1976. An attempt to cultivate mycobacteria in simple synthetic liquid medium containing lecithin-cholesterol liposomes. Jpn J Med Sci Biol 29:109–121. doi:10.7883/yoken1952.29.109 824481

[45] Marrero J, Rhee KY, Schnappinger D, Pethe K, Ehrt S. 2010. Gluconeogenic carbon flow of tricarboxylic acid cycle intermediates is critical for Mycobacterium tuberculosis to establish and maintain infection. Proc Natl Acad Sci USA 107:9819–9824. doi:10.1073/pnas.1000715107 20439709 PMC2906907

[46] Scriba TJ, Penn-Nicholson A, Shankar S, Hraha T, Thompson EG, Sterling D, Nemes E, Darboe F, Suliman S, Amon LM, Mahomed H, Erasmus M, Whatney W, Johnson JL, Boom WH, Hatherill M, Valvo J, Groote MA, Ochsner UA, Aderem A, Hanekom WA, Zak DE. 2017. Sequential inflammatory processes define human progression from M. tuberculosis infection to tuberculosis disease. PLoS Pathog 13:e1006687. doi:10.1371/journal.ppat.1006687 29145483 PMC5689825

[47] Mateos J, Estévez O, González-Fernández Á, Anibarro L, Pallarés Á, Reljic R, Mussá T, Gomes-Maueia C, Nguilichane A, Gallardo JM, Medina I, Carrera M. 2020. Serum proteomics of active tuberculosis patients and contacts reveals unique processes activated during Mycobacterium tuberculosis infection. Sci Rep 10:3844. doi:10.1038/s41598-020-60753-5 32123229 PMC7052228

[48] Parente R, Clark SJ, Inforzato A, Day AJ. 2017. Complement factor H in host defense and immune evasion. Cell Mol Life Sci 74:1605–1624. doi:10.1007/s00018-016-2418-4 27942748 PMC5378756

[49] Herbert AP, Makou E, Chen ZA, Kerr H, Richards A, Rappsilber J, Barlow PN. 2015. Complement evasion mediated by enhancement of captured factor H: implications for protection of self-surfaces from complement. J Immunol 195:4986–4998. doi:10.4049/jimmunol.1501388 26459349 PMC4635569

[50] Kennedy AT, Schmidt CQ, Thompson JK, Weiss GE, Taechalertpaisarn T, Gilson PR, Barlow PN, Crabb BS, Cowman AF, Tham WH. 2016. Recruitment of factor H as a novel complement evasion strategy for blood-stage Plasmodium falciparum infection. J Immunol 196:1239–1248. doi:10.4049/jimmunol.1501581 26700768

[51] Meri T, Amdahl H, Lehtinen MJ, Hyvärinen S, McDowell JV, Bhattacharjee A, Meri S, Marconi R, Goldman A, Jokiranta TS. 2013. Microbes bind complement inhibitor factor H via a common site. PLoS Pathog 9:e1003308. doi:10.1371/journal.ppat.1003308 23637600 PMC3630169

[52] Vogl G, Lesiak I, Jensen DB, Perkhofer S, Eck R, Speth C, Lass-Flörl C, Zipfel PF, Blom AM, Dierich MP, Würzner R. 2008. Immune evasion by acquisition of complement inhibitors: the mould Aspergillus binds both factor H and C4b binding protein. Mol Immunol 45:1485–1493. doi:10.1016/j.molimm.2007.08.011 17915330 PMC5654503

[53] Moore SR, Menon SS, Cortes C, Ferreira VP. 2021. Hijacking factor H for complement immune evasion. Front Immunol 12:602277. doi:10.3389/fimmu.2021.602277 33717083 PMC7947212

[54] Miller DP, Oliver LD Jr, Tegels BK, Reed LA, O’Bier NS, Kurniyati K, Faust LA, Lawson CK, Allard AM, Caimano MJ, Marconi RT. 2016. The Treponema denticola FhbB protein is a dominant early antigen that elicits FhbB variant-specific antibodies that block factor H binding and cleavage by dentilisin. Infect Immun 84:2051–2058. doi:10.1128/IAI.01542-15 27113359 PMC4936362

[55] Riva R, Korhonen TK, Meri S. 2015. The outer membrane protease PgtE of Salmonella enterica interferes with the alternative complement pathway by cleaving factors B and H. Front Microbiol 6:63. doi:10.3389/fmicb.2015.00063 25705210 PMC4319491

[56] Hammerschmidt S, Agarwal V, Kunert A, Haelbich S, Skerka C, Zipfel PF. 2007. The host immune regulator factor H interacts via two contact sites with the PspC protein of Streptococcus pneumoniae and mediates adhesion to host epithelial cells. J Immunol 178:5848–5858. doi:10.4049/jimmunol.178.9.5848 17442969

[57] Agarwal S, Ram S, Ngampasutadol J, Gulati S, Zipfel PF, Rice PA. 2010. Factor H facilitates adherence of Neisseria gonorrhoeae to complement receptor 3 on eukaryotic cells. J Immunol 185:4344–4353. doi:10.4049/jimmunol.0904191 20826755 PMC2944003

[58] Ermert D, Ram S, Laabei M. 2019. The hijackers guide to escaping complement: lessons learned from pathogens. Mol Immunol 114:49–61. doi:10.1016/j.molimm.2019.07.018 31336249

[59] Chen X, Wang J, Wang J, Ye J, Di P, Dong C, Lei H, Wang C. 2023. Several potential serum proteomic biomarkers for diagnosis of osteoarticular tuberculosis based on mass spectrometry. Clin Chim Acta 547:117447. doi:10.1016/j.cca.2023.117447 37353136

[60] Chu Y, Soodeen-Lalloo AK, Huang J, Yang G, Chen F, Yin H, Sha W, Huang X, Shi J, Feng Y. 2019. Sex disparity in severity of lung lesions in newly identified tuberculosis is age-associated. Front Med 6:163. doi:10.3389/fmed.2019.00163 PMC6650771 31380378

[61] Petersen SV, Thiel S, Jensen L, Steffensen R, Jensenius JC. 2001. An assay for the mannan-binding lectin pathway of complement activation. J Immunol Methods 257:107–116. doi:10.1016/s0022-1759(01)00453-7 11687244

[62] Turner MW. 1996. Mannose-binding lectin: the pluripotent molecule of the innate immune system. Immunol Today 17:532–540. doi:10.1016/0167-5699(96)10062-1 8961631

[63] Zhang JX, Gong WP, Zhu DL, An HR, Yang YR, Liang Y, Wang J, Tang J, Zhao WG, Wu XQ. 2020. Mannose-binding lectin 2 gene polymorphisms and their association with tuberculosis in a Chinese population. Infect Dis Poverty 9:46. doi:10.1186/s40249-020-00664-9 32349793 PMC7191747

[64] Kawka M, Brzostek A, Dzitko K, Kryczka J, Bednarek R, Płocińska R, Płociński P, Strapagiel D, Gatkowska J, Dziadek J, Dziadek B. 2021. Mycobacterium tuberculosis binds human serum amyloid A, and the interaction modulates the colonization of human macrophages and the transcriptional response of the pathogen. Cells 10:1264. doi:10.3390/cells10051264 34065319 PMC8160739

[65] Garay-Baquero DJ, White CH, Walker NF, Tebruegge M, Schiff HF, Ugarte-Gil C, Morris-Jones S, Marshall BG, Manousopoulou A, Adamson J, Vallejo AF, Bielecka MK, Wilkinson RJ, Tezera LB, Woelk CH, Garbis SD, Elkington P. 2020. Comprehensive plasma proteomic profiling reveals biomarkers for active tuberculosis. JCI Insight 5:e137427. doi:10.1172/jci.insight.137427 32780727 PMC7526553

[66] Franco Fontes C, Silva Bidu N, Rodrigues Freitas F, Maranhão RC, Santos Monteiro A de S, David Couto R, Martins Netto E. 2023. Changes in serum amyloid A, plasma high-density lipoprotein cholesterol and apolipoprotein A-I as useful biomarkers for Mycobacterium tuberculosis infection. J Med Microbiol 72. doi:10.1099/jmm.0.001726 37389586

[67] Mishra P, Verma VK, Barman L, Sharma J, Gupta P, Mohan A, Arya DS. 2022. Correlation of serum amyloid A1 and interleukin-1beta in response to anti-tubercular therapy. Am J Med Sci 364:316–326. doi:10.1016/j.amjms.2021.12.014 35452629

[68] Schurgers LJ, Cranenburg ECM, Vermeer C. 2008. Matrix Gla-protein: the calcification inhibitor in need of vitamin K. Thromb Haemost 100:593–603. 18841280

[69] Li M, Wang Z, Shao J, Li S, Xia H, Yu L, Hu Z. 2020. Captopril attenuates the upregulated connexin 43 expression in artery calcification. Arch Med Res 51:215–223. doi:10.1016/j.arcmed.2020.02.002 32111501

[70] Murshed M, Schinke T, McKee MD, Karsenty G. 2004. Extracellular matrix mineralization is regulated locally; different roles of two gla-containing proteins. J Cell Biol 165:625–630. doi:10.1083/jcb.200402046 15184399 PMC2172384

[71] Luo G, Ducy P, McKee MD, Pinero GJ, Loyer E, Behringer RR, Karsenty G. 1997. Spontaneous calcification of arteries and cartilage in mice lacking matrix GLA protein. Nature 386:78–81. doi:10.1038/386078a0 9052783

[72] Lu X, Gao B, Liu Z, Tian X, Mao X, Emmanuel N, Zhu Q, Xiao C. 2012. A polymorphism of matrix Gla protein gene is associated with kidney stone in the Chinese Han population. Gene 511:127–130. doi:10.1016/j.gene.2012.09.112 23046575

[73] Sun H, Pan L, Jia H, Zhang Z, Gao M, Huang M, Wang J, Sun Q, Wei R, Du B, Xing A, Zhang Z. 2018. Label-free quantitative proteomics identifies novel plasma biomarkers for distinguishing pulmonary tuberculosis and latent infection. Front Microbiol 9:1267. doi:10.3389/fmicb.2018.01267 29951049 PMC6008387

[74] Ahmadi F, Ghadiri A, NashibI R, Roozbeh F, Alizadeh-Navaei R. 2017. Serum mannan-binding lectin in patients with pulmonary tuberculosis: Its lack of a relationship to the disease and response to treatment. Med J Islam Repub Iran 31:66. doi:10.14196/mjiri.31.66 29445695 PMC5804467

[75] García-Gasalla M, Milá Llambí J, Losada-López I, Cifuentes-Luna C, Fernández-Baca V, Pareja-Bezares A, Mir-Villadrich I, Payeras-Cifré A. 2014. Mannose-binding lectin exon 1 and promoter polymorphisms in tuberculosis disease in a Mediterranean area. Int J Immunogenet 41:306–311. doi:10.1111/iji.12126 24910008

[76] Selvaraj P, Jawahar MS, Rajeswari DN, Alagarasu K, Vidyarani M, Narayanan PR. 2006. Role of mannose binding lectin gene variants on its protein levels and macrophage phagocytosis with live Mycobacterium tuberculosis in pulmonary tuberculosis. FEMS Immunol Med Microbiol 46:433–437. doi:10.1111/j.1574-695X.2006.00053.x 16553818

