# Supplementary material for: Proteomic analysis of plasma unravels dynamic pathways and potential biomarkers indicating disease stages following Mtb infection
Source: mSystems. 2025 Jul 30;10(8):e00616-25. doi: 10.1128/msystems.00616-25 (PMC12363209; doi:10.1128/msystems.00616-25)
Supplement: Supplemental Figures — Figures S1 to S4. [file msystems.00616-25-s0001.docx]

**Supplementary Figures**

**Figure S1.** Overview of plasma proteome data obtained by 4D-DIA method. (A) Bar plot shows the counts of identified proteins in each sample. Bars are colored by disease stages. (B) Bar plot outlines the protein coverage and the number of corresponding proteins. (C) Distribution chart delineates the molecular weight of detected proteins. (D) Chart shows the length distribution of identified peptides. (E) The density and cumulative proportion of proteins against relative abundance. Green represents proteins with missing values, and red represents those with valid data. (F) Curve plot features the abundance distribution of original data and data with missing values imputed by random forest method. Lines are colored by disease stages. (G) Jitter diagram and boxplot delineate the coefficient of variation (CV) of protein abundance in HC, LTBI, and ATB. Points are colored according to the three conditions. Between-group comparisons of CV are performed by Wilcoxon test, with *P*-values adjusted using Bonferroni method.

**Figure S2.** Venn’s diagram showing the number of DEPs shared between groups.

**Figure S3.** Identification of protein modules associated TB stages. (A) Dendrogram presents the similarity among samples, with trait heatmap denoting disease stages. (B) Changes in the scale-free topology model fitting index and mean connectivity of network as soft‐thresholding power increases. The power at which the network is scaled to have a fitting index >0.85 is used to construct co-expression networks.

**Figure S4.** ROC curves of 11 DEPs from validation cohort in discriminating ATB from LTBI.


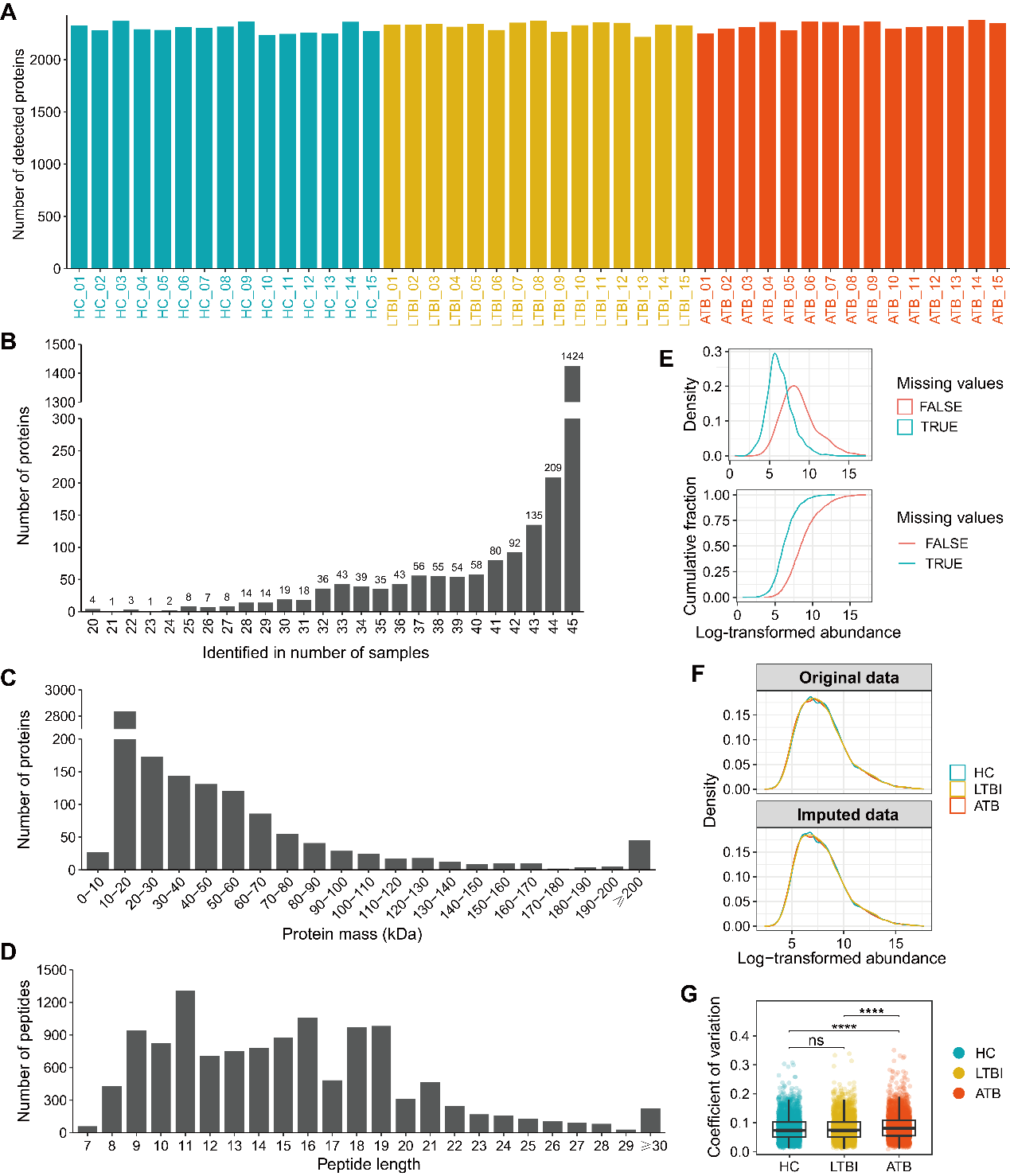


**Figure S1.** Overview of plasma proteome data obtained by 4D-DIA method. (A) Bar plot shows the counts of identified proteins in each sample. Bars are colored by disease stages. (B) Bar plot outlines the protein coverage and the number of corresponding proteins. (C) Distribution chart delineates the molecular weight of detected proteins. (D) Chart shows the length distribution of identified peptides. (E) The density and cumulative proportion of proteins against relative abundance. Green represents proteins with missing values, and red represents those with valid data. (F) Curve plot features the abundance distribution of original data and data with missing values imputed by random forest method. Lines are colored by disease stages. (G) Jitter diagram and boxplot delineate the coefficient of variation (CV) of protein abundance in HC, LTBI, and ATB. Points are colored according to the three conditions. Between-group comparisons of CV are performed by Wilcoxon test, with *P*-values adjusted using Bonferroni method.


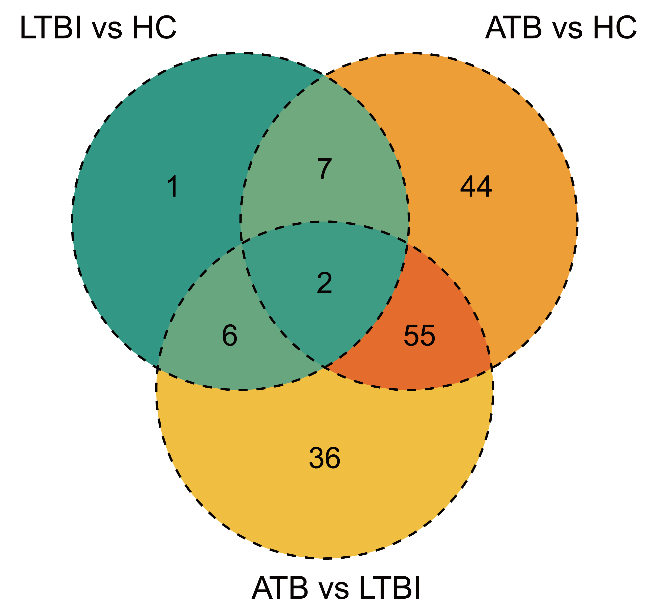


**Figure S2.** Venn’s diagram showing the number of DEPs shared between groups.


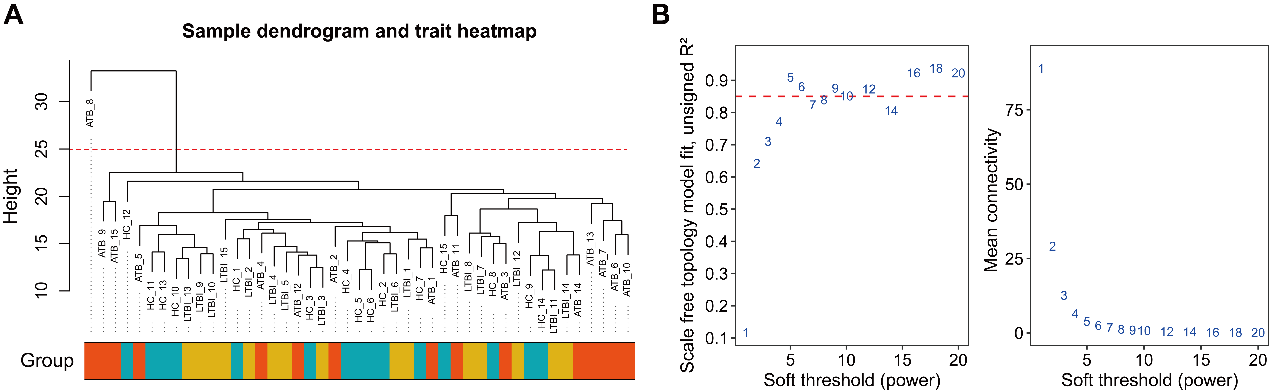


**Figure S3.** Identification of protein modules associated TB stages. (A) Dendrogram presents the similarity among samples, with trait heatmap denoting disease stages. (B) Changes in the scale-free topology model fitting index and mean connectivity of network as soft‐thresholding power increases. The power at which the network is scaled to have a fitting index >0.85 is used to construct co-expression networks.


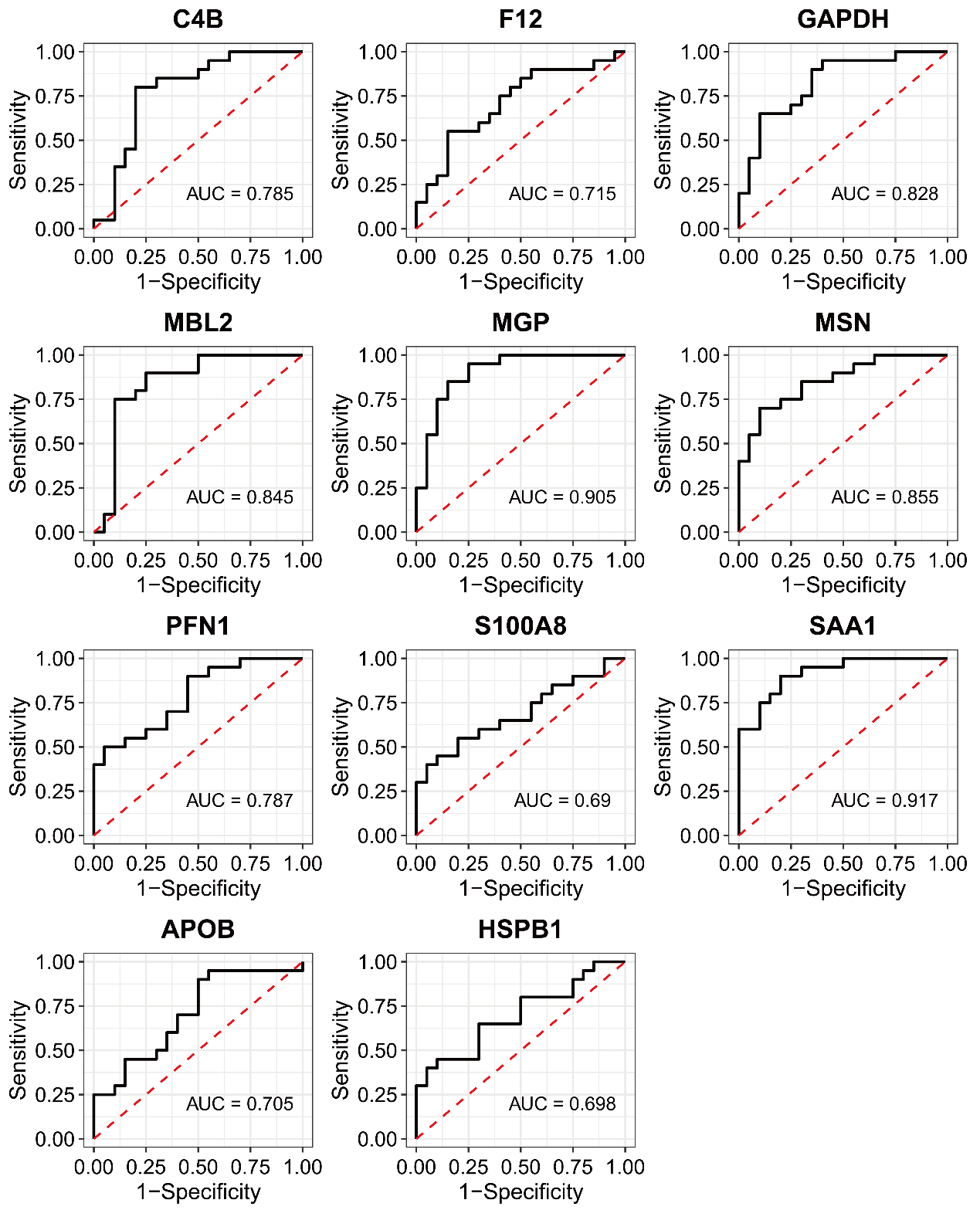


**Figure S4.** ROC curves of 11 DEPs from validation cohort in discriminating ATB from LTBI.
